# Gemcitabine, lycorine and oxysophoridine inhibit novel coronavirus (SARS-CoV-2) in cell culture

**DOI:** 10.1080/22221751.2020.1772676

**Published:** 2020-06-02

**Authors:** Ya-Nan Zhang, Qiu-Yan Zhang, Xiao-Dan Li, Jin Xiong, Shu-Qi Xiao, Zhen Wang, Zhe-Rui Zhang, Cheng-Lin Deng, Xing-Lou Yang, Hong-Ping Wei, Zhi-Ming Yuan, Han-Qing Ye, Bo Zhang

**Affiliations:** a Key Laboratory of Special Pathogens and Biosafety, Wuhan Institute of Virology, Center for Biosafety Mega-Science, Chinese Academy of Sciences, Wuhan, People’s Republic of China; b The Joint Center of Translational Precision Medicine, Guangzhou Institute of Pediatrics, Guangzhou Women and Children’s Medical Center, Guangzhou, People’s Republic of China; c The Joint Center of Translational Precision Medicine, Wuhan Institute of Virology, Chinese Academy of Sciences, Wuhan, People’s Republic of China; d School of Medicine, Hunan Normal University, Changsha, People’s Republic of China; e University of Chinese Academy of Sciences, Beijing, People’s Republic of China; f Drug Discovery Center for Infectious Disease, Nankai University, Tianjin, People’s Republic of China

**Keywords:** Novel coronavirus, alkaloid, broad-spectrum antiviral, 2019-nCoV, SARS-CoV-2

## Abstract

The emerging SARS-CoV-2 infection associated with the outbreak of viral pneumonia in China is ongoing worldwide. There are no approved antiviral therapies to treat this viral disease. Here we examined the antiviral abilities of three broad-spectrum antiviral compounds gemcitabine, lycorine and oxysophoridine against SARS-CoV-2 in cell culture. We found that all three tested compounds inhibited viral replication in Vero-E6 cells at noncytotoxic concentrations. The antiviral effect of gemcitabine was suppressed efficiently by the cytidine nucleosides. Additionally, combination of gemcitabine with oxysophoridine had an additive antiviral effect against SARS-CoV-2. Our results demonstrate that broad-spectrum antiviral compounds may have a priority for the screening of antiviral compounds against newly emerging viruses to control viral infection.

Since the late 2019 when a cluster of pneumonia associated with the emerging novel coronavirus, SARS-CoV-2 was reported in Wuhan, China. Since then, the outbreak has spread rapidly and developed into a global pandemic. At the time the paper was submitted, about 210 countries and territories around the world have reported more than 2.9 million confirmed cases of SARS-CoV-2 including more than 203,289 deaths (https://www.worldometers.info/coronavirus/#countries). Currently, there is no antiviral therapy or vaccine available for human usage. It is urgent to develop some antiviral compounds for the treatment of SARS-CoV-2 infection because of its high infectivity and morbidity and its ability to cause epidemics worldwide. Different compounds, such as remdesivir and chloroquine have been reported to inhibit SARS-CoV-2 replication effectively *in vitro* [1]. The efficacy of these drugs for SARS-CoV-2 is still under investigation by clinical experiments.

In this study, we tested antiviral activity of gemcitabine, lycorine and oxysophoridine against SARS-CoV-2 infection in cell culture, and chloroquine was used as a positive control [1]. Vero-E6 cells infected with SARS-CoV-2 (WIV04) [2] at a multiplicity of infection (MOI) of 0.005 were treated with increased concentrations of compounds. The viral RNAs in cell culture media were quantified with quantitative real-time RT-PCR (qRT-PCR). All these three compounds exhibited dose-dependent inhibition of 2019-CoV replication in infected cells as chloroquine (Figure 1(A)). The EC_50_ values of gemcitabine, lycorine, oxysophoridine and chloroquine were 1.24, 0.31, 0.18 and 1.36 μM, respectively. To confirm that the inhibition of viral replication was not due to compound-mediated cytotoxicity, a cell proliferation-based cytotoxicity assay was performed. As shown in Figure 1(A), the CC_50_ (50% cytotoxic concentration) values were all above 40 μM. The selectivity index (SI[CC_50_/EC_50_]) in Vero cells were above 33, 129, 222 and 30, respectively. We then performed indirect immunofluorescence assay (IFA) for viral protein expression using anti-NP antibody to further verify the antiviral activity of all the three compounds. The number of IFA positive cells indicated the capability of virus replication and spreading within infected cells. Comparing with DMSO treated group, compounds treated groups showed significantly decreased positive cells with increased concentrations of compounds (Figure 1(B)). Consistently, the inhibitory effects of these compounds on SARS-CoV-2 replication were also displayed through dose-dependent rescuing CPE in infected cells (Figure 1(C)). Additionally, all three compounds could efficiently inhibit SARS-CoV-2 in Huh-7 cells (Figure 1(D)), which indicated that their antiviral activities were not cell type dependent. Given the successful application of antiviral combination therapy for treatment of HIV and enterovirus infection, the antiviral effect of gemcitabine in combination with increased concentrations of oxysophoridine was also evaluated in Vero-E6 cells (Figure 1(E)). The expected additive inhibitory effects were observed with the combination of gemcitabine and oxysophoridine.

**Figure 1.**
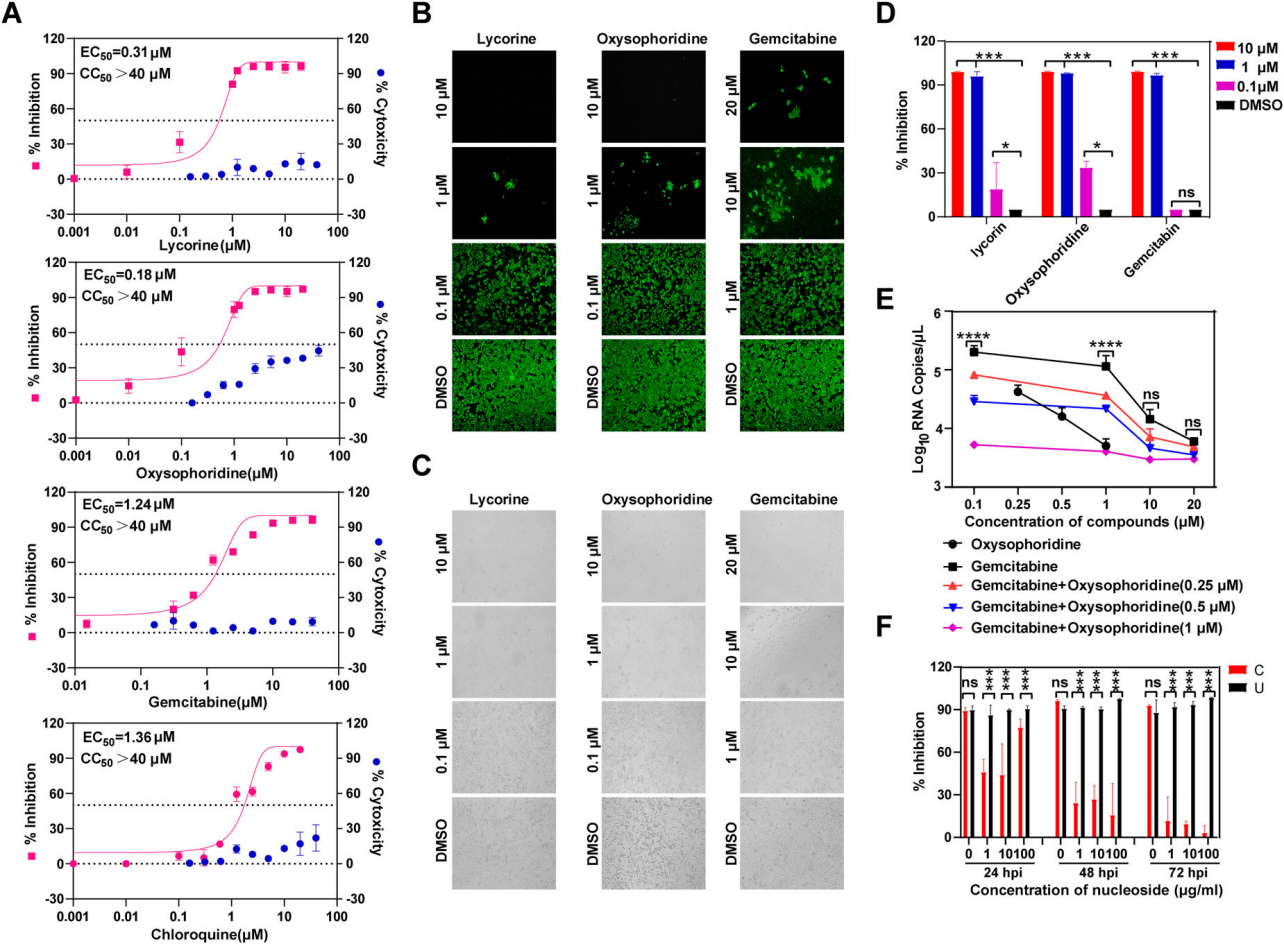
The antiviral activities of gemcitabine, lycorine and oxysophoridine against SARS-CoV-2 in cell culture. (A) Dose-dependent inhibition of gemcitabine, lycorine and oxysophoridine on SARS-CoV-2 infectivity. Vero-E6 cells infected with SARS-CoV-2 at an MOI of 0.005 were treated with increasing concentration of compounds for 24 hours. Real-time RT-PCR was used to quantify viral RNA copy numbers in culture media. The cytotoxicity of the compounds on Vero-E6 cells was examined with CCK-8 assay kit. Antiviral activity and cytotoxicity are shown in pink and blue, respectively. The EC_50_ and CC_50_ are displayed in the upper right corner for each compound. At least three independent experiments were performed, and one representative experiment was presented. The data were represented as mean ± standard deviation (SD) of the triplicate measurements. (B) IFA analysis of the inhibition of the compounds on SARS-CoV-2 replication. At 24 hpi, the infected cells treated with different concentration of compounds were fixed and subjected to IFA using the primary antibody against NP protein of SARS-CoV-2. (C) Characterization of antiviral activities based on CPE-reduction assay. At 48 hpi, the CPE in infected Vero-E6 cells was visualized under light microscope. (D) Dose-dependent inhibition of gemcitabine, lycorine and oxysophoridine on SARS-CoV-2 infectivity in Huh-7 cells. The cells infected with SARS-CoV-2 at an MOI of 0.001 were treated with increasing concentration of compounds for 72 hours, and viral RNA copy numbers in culture media were quantified through real-time RT-PCR. The asterisks denote statistical differences between indicated groups.*p < 0.1, **p < 0.01, ***p < 0.001, ****p < 0.0001 (One-way ANOVA with Kruskal-Wallis test). n.s. indicates no statistical differences. (E) Additive anti-SARS-CoV-2 effect of gemcitabine in combination with different concentrations of oxysophoridine. The asterisks denote statistical differences between indicated groups.*p < 0.1, **p < 0.01, ***p < 0.001, ****p < 0.0001(Two-way ANOVA). n.s. indicates no statistical differences. (F) Suppression of the antiviral activity of gemcitabine by cytidine. Vero-E6 cells were infected with SARS-CoV-2 and simultaneously treated with 10 μM gemcitabine plus different concentrations of cytidine or uridine. The asterisks denote statistical differences between indicated groups.*p < 0.1, **p < 0.01, ***p < 0.001, ****p < 0.0001 (Student’s *t*-test). n.s. indicates no statistical differences. All above experiments were performed at least three independent, and one representative experiment was presented. All the Data were represented as mean ± standard deviation (SD).

Gemcitabine is a cytidine analog approved by the FDA for the treatment of various cancers [3]. Increasing evidence has shown that gemcitabine is an effective broad spectrum antiviral agent against multiple RNA viruses which include Middle East respiratory syndrome coronavirus (MERS-CoV), severe acute respiratory syndrome coronavirus (SARS-CoV) [4], Zika virus [5], influenza virus [6] and enterovirus. Gemcitabine inhibits both MERS-CoV and SARS-CoV with micromolar EC50s (1.22 μM and 4.96 μM, respectively) [4], which are similar to the EC_50_ of SARS-CoV-2 (1.24 μM) in our study. It is thought that gemcitabine might exert its antiviral activities by targeting the salvage pathway of pyrimidine biosynthesis and stimulating innate immunity, at least in the cases of enterovirus [7] and influenza virus [6]. To examine whether it acts by the same antiviral mechanism against SARS-CoV-2, we added additional natural nucleosides (C and U) with gemcitabine in cell culture. Similar to the previous results observed in enterovirus [7], the addition of exogenous cytidine significantly inhibited the antiviral activity of gemcitabine against SARS-CoV-2 (Figure 1(F)). Our results thus indicated that gemcitabine may inhibit SARS-CoV-2 replication through the modulation of nucleotide biosynthesis, the same mechanism as did in enterovirus [7]. Although we are unable to draw definitive conclusions regarding the efficacy of gemcitabine against SARS-CoV-2 due to the lack of *in vivo* animal data, its antiviral efficiencies have been demonstrated in the mice models of enterovirus and human immunodeficiency virus (HIV) [7]. Additionally, the mean peak plasma concentration of gemcitabine observed in patients with advanced non-small cell lung cancer (NSCLC) could reach 17 µM after bronchial artery infusion or intravenous infusion [8], which is much higher than EC_50_ of 1.21 μM in Vero-E6 cell. Altogether, these results support the therapeutic potential of gemcitabine as effective antivirals with low toxicity against SARS-CoV-2. Further *in vivo* evaluation about the antiviral activity of gemcitabine against SARS-CoV-2 infection is going to be carried out in the near future, which will accelerate its application in clinical trials.

Different with gemcitabine, both oxysophoridine and lycorine are bioactive alkaloids derived from Chinese herbal medicines. Alkaloids are a group of naturally occurring chemical compounds that contain mostly basic nitrogen atoms [9]. Oxysophoridine is an alkaloid extracted from Sophora alopecuroides Linn. Many studies based on *in vitro* cellular or *in vivo* animal assays have shown that oxysophoridine has various pharmacological activities, including suppression of the growth of hepatocellular carcinoma [10] and colorectal cancer cells [11] by regulating apoptosis associated with the Bcl-2/Bax/caspase-3 signalling pathway, and alleviation of spinal cord injury via anti-inflammatory, anti-oxidative stress and anti-apoptosis effects [12]. In the present study, we found a new indication for oxysophoridine as a compound inhibiting SARS-CoV-2 infection in cell culture. To our knowledge, this is the first time to report the antiviral activity of oxysophoridine. We also found that oxysophoridine could efficiently inhibit flavivirus and alphavirus replication (unpublished data), which indicated that oxysophoridine may have a broad-spectrum antiviral activity against RNA viruses.

Lycorine is an active alkaloid abundant in Amaryllidaceae with a wide range of biological functions for cancer and infectious diseases treatment [13]. Unlike oxysophoridine, lycorine has been well known to exhibit antiviral activities against enterovirus, flaviviruses, HIV-1, SARS-CoV and hepatitis C virus. Regarding the exact antiviral mechanism of lycorine, however, it remains elusive. Different drug resistant mutations of lycorine have been identified within the viral protease of enterovirus or nonstructural protein 2 K of West Nile virus. Meanwhile, it was reported that lycorine was able to (i) inhibit the export of influenza virus nucleoprotein from the nucleus [14], and (ii) downregulate autophagy [15] or block the elongation of viral RNA translation during EV71 infection [13] to suppress viral replication. Altogether, we speculate that the mechanism behind the anti-SARS-CoV-2 activity of lycorine is probably attributed to modulating host factors instead of directly targeting viral factors.

Overall, more information is needed regarding anti-SARS-CoV-2 issues of both oxysophoridine and lycorine including antiviral mechanisms, the safety profile and toxicological evaluation in future study.
